# RUNX3, EGR1 and SOX9B Form a Regulatory Cascade Required to Modulate BMP-Signaling during Cranial Cartilage Development in Zebrafish

**DOI:** 10.1371/journal.pone.0050140

**Published:** 2012-11-27

**Authors:** Julia Dalcq, Vincent Pasque, Aurélie Ghaye, Arnaud Larbuisson, Patrick Motte, Joseph A. Martial, Marc Muller

**Affiliations:** 1 Laboratory for Molecular Biology and Genetic Engineering, GIGA-R, Université de Liège, Liège, Belgium; 2 Plant Functional Genomics and Molecular Imaging and Center for Assistance in Technology of Microscopy, University of Liège, Liège, Belgium; University of Colorado, Boulder, United States of America

## Abstract

The cartilaginous elements forming the pharyngeal arches of the zebrafish derive from cranial neural crest cells. Their proper differentiation and patterning are regulated by reciprocal interactions between neural crest cells and surrounding endodermal, ectodermal and mesodermal tissues. In this study, we show that the endodermal factors Runx3 and Sox9b form a regulatory cascade with Egr1 resulting in transcriptional repression of the *fsta* gene, encoding a BMP antagonist, in pharyngeal endoderm. Using a transgenic line expressing a dominant negative BMP receptor or a specific BMP inhibitor (dorsomorphin), we show that BMP signaling is indeed required around 30 hpf in the neural crest cells to allow cell differentiation and proper pharyngeal cartilage formation. Runx3, Egr1, Sox9b and BMP signaling are required for expression of *runx2b*, one of the key regulator of cranial cartilage maturation and bone formation. Finally, we show that e*gr1* depletion leads to increased expression of *fsta* and inhibition of BMP signaling in the pharyngeal region. In conclusion, we show that the successive induction of the transcription factors Runx3, Egr1 and Sox9b constitutes a regulatory cascade that controls expression of Follistatin A in pharyngeal endoderm, the latter modulating BMP signaling in developing cranial cartilage in zebrafish.

## Introduction

In vertebrates, major parts of the craniofacial skeleton derive from cranial neural crest cells (cNCCs) that previously migrated into the pharyngeal arches [1]. cNCCs form bilaterally at the dorsal edge of the neural tube by the end of gastrulation. Later, they lose their adhesive properties, separate from the neural tube and migrate ventrally to finally give rise to cartilage, neurons, glial cells and pigment cells. Based on their original antero-posterior position along the posterior brain, cNCCs exhibit a segmental organization and specification to form three clusters of cells that migrate and separate into the pharyngeal arches [1]. Each pharyngeal arch is formed by a mesoderm-derived core embedded in the neural crest-derived cartilage precursor cells and encased on the medial side by endoderm and on the lateral side by ectoderm.

Differentiation of cNCCs into mature chondrocytes is controlled by a number of transcription factors. In zebrafish, pre-migratory cNCCs express *tfap2α* starting at 12 hours post fertilization (hpf). *tfap2α* mutants show defects both in pre-migratory cNCC specification and in cartilage development [2]. Tfap2α controls expression of the transcription factor Dlx2a, which is strictly required for correct cNCC migration and survival [3], [4]. Similar to Sox9 in mammals, its co-orthologs Sox9a and Sox9b are known key regulators of cartilage development in zebrafish. Sox9 stimulates expression of the cartilage-specific collagen type 2 gene *Col2a1* in mouse (Bell, Leung 1997, Lefebvre 1997), while in zebrafish both *sox9a* and *sox9b* are required for *col2a1* expression. However, they play both distinct and overlapping functions in zebrafish chondrogenesis [5]. Both factors are expressed in migratory cranial neural crest cells (cNCC) without influencing migration of *dlx2*-expressing cNCC. After migration of the cNCCs into the pharyngeal arches, *sox9a* is expressed in cNCCs under control of *dlx2a* [4] while s*ox9b* is only expressed in the pharyngeal endoderm [5]. Mutation of *sox9a* in the *jef* mutant leads to a complete absence of cartilage [6], while *sox9b* mutation causes an absence of ceratobranchials and a reduction of the two most anterior pairs of arches. Sox9a is essential for endochondral ossification, while sox9b has an additional role in formation of some dermal bones [5]. In mammals, Sox9 is essential for *Runx2b* expression [7], [8], but in zebrafish, only Sox9b has this role. Runx2 is an essential transcription factor for osteoblast and early chondrocycte differentiation [9] as well as cartilage and bone development in vertebrates.

In addition to these endogenous patterning cues, cNCCs also receive external signals from surrounding cells and extracellular matrix, such as Fgf, BMP and Shh signals [10], [11], [12], [13], which are absolutely required for pharyngeal cartilage differentiation, segmentation, and cell survival. Recently, BMP signaling was shown to promote ventral arch development just after cNCC migration, however a later function for BMPs in craniofacial skeleton patterning was also shown [14]. Only the early action takes place upstream and in parallel to Endothelin 1 signaling. In zebrafish, lack of pharyngeal endoderm leads to an absence of pharyngeal cartilage [15], [16]. Homozygous *casanova* (*cas*) and *bonny and clyde* (*bon*) mutants lack endodermal markers and present severe cartilage defects [17], [18], [19], [20]. *van gogh* (vgo) mutants make pharyngeal endoderm but fail to form pouches, which leads to an absence of cNCC segmentation into distinct elements [21]. Transplantation of wild-type endoderm into *vgo* and *cas* mutants restores the cartilaginous phenotype [21]. Thus, endoderm controls the fate of chondrogenic cNCC and has a cell non-autonomous action on pharyngeal cartilage.

The early growth response (EGR) family contains four highly conserved zinc finger (C2H2 type) transcription factors that mediate the cellular response to many stimuli inducing *e.g.* mitosis, differentiation and apoptosis [22]. In mice, *Egr1* is expressed during embryogenesis in cartilage, bones, teeth, salivary glands, nasal glands, developing vibrissa, tendons and skeletal muscles [23]. In zebrafish, the 3.4 kb long *egr1* gene is composed of two exons and one intron [24]; no transcript was detected by *in situ* hybridization before gastrulation [25]. Starting at somitogenesis, mRNA was observed in posterior adaxial cells of the presomitic mesoderm until 23 hpf. Various expression domains were also detected in different brain regions and in the eye. Around 30 hpf, e*gr1* expression was observed in the pharyngeal region until at least 48 hpf.

Egr1 is known to play various roles such as tumor promotion in prostate [26], [27], tumor suppressor in various cancers [28], [29], cell growth and differentiation [30] and hematopoietic cell maturation [31]. *Egr1* knockout mice display sterility for both sexes and are smaller due to defects in adenohypophysis development and particularly LHβ expression [32], [33], [34]. Egr1 was also shown to play a role in osteoblast differentiation [35] and is induced in osteoblasts during microgravity simulation and after mechanical strain [36]. In rat bone marrow cells, Egr1 was shown to act as a suppressor of osteoclastogenesis [37]. *Egr1* KO mice present increased bone resorption, a reduced bone mass and an increased production of the osteoclastogenic cytokine M-CSF (macrophage Colony-Stimulating Factor) in stromal cells of the bone matrix [38], [39], [40]. Egr1 is down-regulated in osteoarthritic cartilage compared to normal tissue [41] and is required for TNFα regulation in chondrocytes of catabolic and anabolic genes for the cartilage extracellular matrix [42]. In zebrafish, knock down of *egr1* leads to a reduction of the eye size, while the retina and lens lack appropriate differentiation [43].

In this study, we report that the transcription factor Egr1 plays a specific role in pharyngeal cartilage formation during zebrafish embryogenesis, while it is expressed in pharyngeal endoderm. We show that Egr1 is part of a regulatory cascade, together with the endodermal factors Runx3 and Sox9b, that suppresses expression of follistatin A (*fsta*), a known BMP antagonist. We also show that BMP signaling is required around 30 hpf in the neural crest cells for *runx2b* expression in chondrocytes and proper pharyngeal cartilage formation, while BMP signaling is absent in the pharyngeal region upon *egr1* knock-down.

## Materials and Methods

### Fish and Embryo Maintenance

Zebrafish (*Danio rerio*) were reared in a recirculating system from Techniplast, Italy at a maximal density of 7 fish/l. The water characteristics were as follows: pH = 7.4, conductivity = 500 µScm-1, temperature = 28°C. The light cycle was controlled (14 h light, 10 h dark). Fish were fed twice daily with dry powder (ZM fish food®) with size adapted to their age, and once daily with fresh *Artemia salina* nauplii (ZM fish food®). Larvae aged less than 14 days were also fed twice daily with a live paramecia culture. Wild type embryos from the AB strain were used and staged according to Kimmel [44]. The transgenic line *Tg(hsp70l:dnBmpr-GFP)* [45] was obtained from the ZIRC (Eugene, Oregon, USA).

Breeding: the day before breeding, 2 males and 2 females were placed in breeding tanks out of the recirculating system, with an internal divider to prevent unwanted mating. On the day of breeding, fish were placed in fresh aquarium water and the divider was removed to allow mating. Eggs were collected every 30 minutes and raised in E3 (5 mM Na Cl, 0.17 mM KCl, 0.33 mM CaCl_2_, 0.33 mM MgSO_4_, 0.00001% Methylene Blue).

All experiments and the entire study were evaluated by the Ethical Committee of the University of Liege, Belgium and accepted under the file numbers 377, 568 and 1074.

### Loss of Function and Rescue Experiments

Morpholino oligonucleotides (MO) were synthesized by Gene Tools (Philomath, OR, USA) and are complementary to the 5′ sequence near the translation start site or to the splice junctions. MO stock solutions were prepared as suggested by Gene Tools. Tetramethylrhodamine dextran (Invitrogen, Belgium) was added at a concentration of 0.5% to sort correctly injected embryos a few hours after injection. The morpholino sequences are as follow:

MO splicing *egr1*: GGATTTAGTGCTTACCTCCAGCAAG.

MO translation *egr1*: TGCAGCCATCTCTCTGGAGTGTGCT.

MO translation *runx3*: TGCTCGGGTCTACGGGAATATGCA [46].

MO translation *fst1a*: CTGACGTTTAGCATCCTTAGCATG [47].

MO standard control: CCTCTTACCTCAGTTACAATTTATA (Gene-Tools®).

An e*gr1* cDNA fragment starting at the ATG start codon and spanning the entire coding region was obtained by PCR-amplification from the cDNA clone [25] and inserted into pCRII-TOPO and a SV40 polyadenylation signal sequence was added at the 3′ end. *egr1* mRNA was synthesized using mMessage mMachine Sp6 (Ambion®) and injected alone or co-injected with a morpholino at the one-cell stage with a microinjector (Narishige®).

Efficiency of the splicing morpholino MO*egr1* spl was examined by RT-PCR using SuperScript® from Invitrogen (Gent, Belgium). mRNA of 50 injected embryos was extracted for each experiment. The primers used were: Zf-Egr1 forward: 5′-CAGTTTGATCACCTTGCTGG-3′; Zf-Egr1 reverse: 5′-GGAAGACGTGGAAGAGGAAG-3′.

### Whole-mount *in situ* Hybridization and Immunohistochemisrty

Injected embryos for *in situ* hybridization were raised in presence of 0.003% of 1-phenyl-2-thiourea until the desired stage, fixed overnight in 4% of PFA at 4°C and stored in 100% methanol at −20°C until use. Visible *in situ* hybridizations (ISH) were performed as described [48]. Fluorescent labeling was performed as described [49], [50].

Whole-mount fluorescent immunohistochemistry was described in [51]. We used the following antibodies: polyclonal rabbit anti-phospho-Smad (1∶200, Cell Signaling®), polyclonal mouse anti-GFP (1∶250, Roche®) and fluorescently conjugated Alexa antibodies (Molecular Probes®).

### Image Acquisition

Pictures of visible *in situ* hybridization were taken by Nikon® Eclipse 90i microscope and NIS-Elements microscope imaging software. Fluorescent images were acquired with a Leica® SP2 confocal microscope.

### Alcian Blue Staining

Cartilage was stained with Alcian Blue 8 GX (Sigma®). Four days old embryos were fixed in PFA 4% for 2h at room temperature, rinsed with PBST and finally stained overnight with 10 mM Mg Cl2/80% ethanol/0.04% Alcian Blue solution. Embryos were rinsed with 80% ethanol/10 mM MgCl2. Pigments were bleached in H2O2 3%/KOH 0,5% for 1 h. 25% glycérol/0,1% KOH, 50%Glycerol/0,1% KOH.

### Heat Shock Conditions

Transgenic embryos *hsp70l:dnBmpr-GFP* [45] were heat shocked at desired stages by placing them into a water bath for 30 minutes at 37°C and afterward placed back at 28°C. Two hours after the heat shock, the embryos were screened for GFP fluorescence. Embryos not expressing GFP were used as non-transgenic controls.

### Dorsomorphin Treatments

10 mM stock solution of dorsomorphin (Calbiochem®) was diluted in DMSO (Calbiochem®). Embryos at desired stage were placed into multiwell plates with dorsomorphin diluted in E3 rearing medium at the desired concentration during a specific time period. DMSO alone was used as control. Embryos were then rinsed several times with E3, raised in E3 and finally fixed at the desired stage.

## Results

### Egr1 is Essential for Zebrafish Pharyngeal Cartilage Development

**Figure 1:**
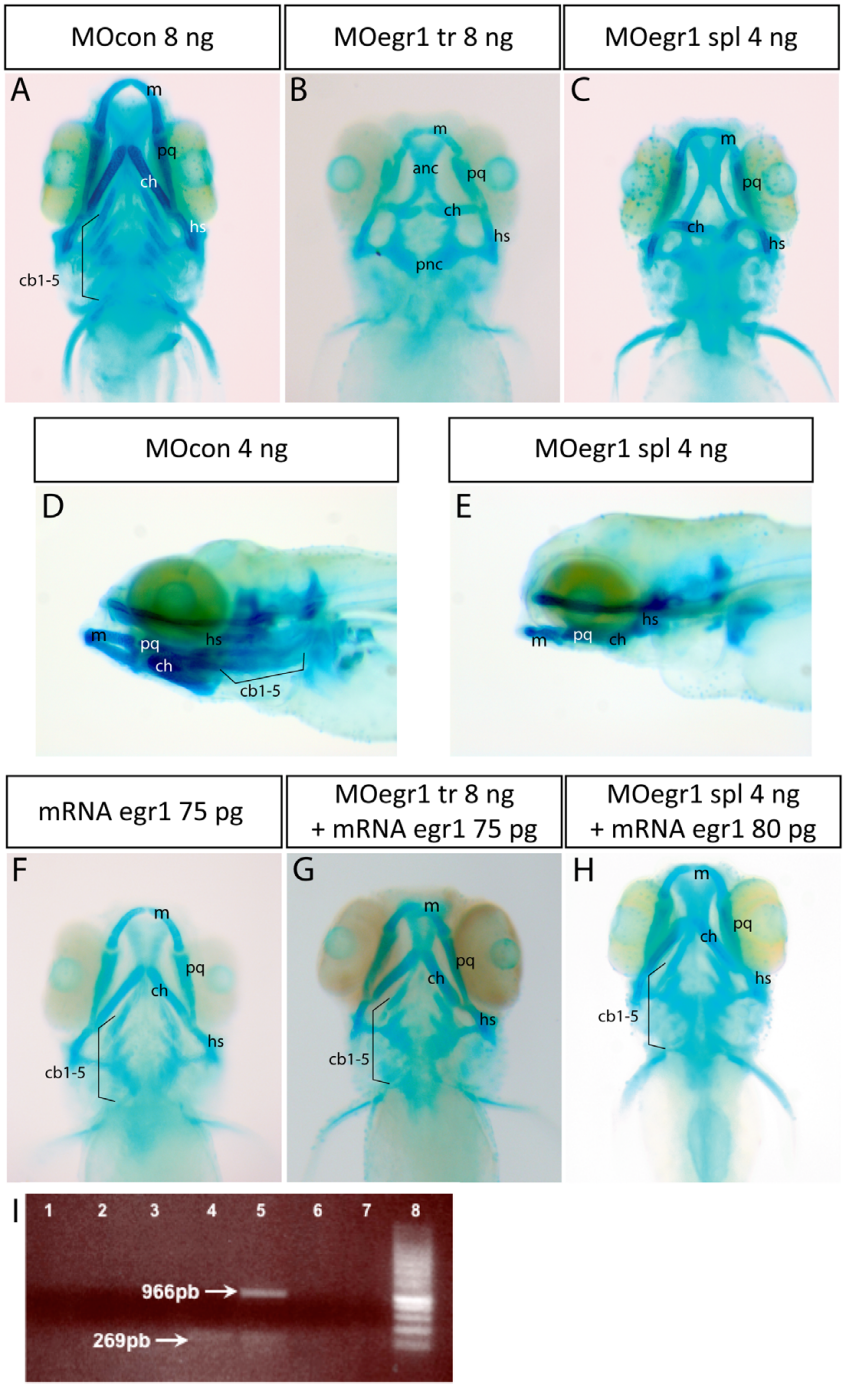
Knock-down of *egr1* severely affects head cartilage formation at 4 dpf. (A–I) Head cartilages were stained with Alcian Blue in morpholino treated larvae at 4 dpf; ventral (A–C) and lateral (D,E) views are shown. (A,D) control 8 ng MOcon treated larvea. (B) 8 ng translation MOegr1 injected larveas display an absence of ceratobranchials and a reduction of size and mis-shaping of pharyngeal cartilage compared to controls (A); (C,E) 4ng splicing MOegr1 injected embryos display similar cartilage defects than 8 ng translation MOegr1 (B). (F) Ectopic expression of Egr1 does not significantly affect cartilage development. (G) Rescue of 8 ng MOegr1 tr treated larvae restores all cartilaginous elements of the viscerocranium. (H) A complete restoration of all cartilage elements is obtained by rescuing 4 ng splicing MOegr1 injected larvae. Meckel’s cartilage (m), palatoquadrate (pq), ceratohyal (ch) and hyosymplectic (hs), ceratobranchials 1 to 5 (cb1-5). (I) Agarose gel electrophoresis analysis of RT-PCR products from mRNA of injected embryos: 1) control mRNA; 2) mRNA of embryos injected with MOcon 4ng and without reverse transcriptase; 3) mRNA of embryos injected with MOegr1 spl 4 ng and without reverse transcriptase; 4) cDNA of embryos injected with MOcon 4 ng. Presence of a band at 269 bp, intron has been spliced properly; 5) cDNA of embryos injected with MOegr1 spl 4 ng. Presence of a band at 966 bp indicating that intron has not been properly spliced. However a residual band at 269 bp reveals that the mRNA has been partially spliced; 6) and 7) cDNA of MOcon 4 ng and MOegr1 spl 4 ng injected embryos that have not undergone the PCR step; 8) molecular weight marker.

To gain insights into an embryonic role of Egr1 in cartilage formation, we depleted Egr1 in developing zebrafish embryos by microinjecting fertilized embryos with morpholinos (MO) directed against *egr1* transcripts or against a control sequence (MOcon). Two different morpholinos against *egr1* were used, one inhibiting *egr1* splicing (MOegr1 spl) and the other preventing translation of *egr1* mRNA (MO egr1 tr). Injection of MO egr1 spl efficiently inhibited egr1 splicing in embryos, as judged by RT-PCR of 2 days post fertilization (dpf) embryos (Fig.1I), whereas injection of MOcon did not.

Pharyngeal and cranial cartilages were stained with Alcian Blue in 4 dpf embryos. Remarkably, in embryos injected with 8 ng of MOegr1 tr, branchial cartilages were completely absent, while Meckel’s cartilage and the palatoquadrate were significantly reduced and the ceratohyal and hyosymplectic misshapen (104/119; 89%) (Fig.1A,B). A similar phenotype was obtained when 4 ng MO egr1 spl was injected, with 87% (120/137) of injected individuals failing to develop branchial cartilages and displaying reduction and malformation of the two first pharyngeal arches (Fig.1C). Axis modification of the hyosimplectics and ceratohyals was also observed in a lateral view (Fig.1B,C,D,E). These results indicate that egr1 may be required for craniofacial development in zebrafish.

To test the specificity of the observed phenotype, we co-injected *egr1* mRNA together with MO egr1 or MOcon. Co-injection of 75 pg of egr1 mRNA together with 8 ng MOegr1 tr rescued the development of pharyngeal and cranial skeleton in 87% of co-injected embryos (43/49; 87%) (Fig.1G), while branchial arch development in 4 ng MOegr1 spl injected embryos was also rescued by *egr1* mRNA expression (Fig.1H). In contrast, injection of 75 pg egr1 mRNA alone had only slight effects on formation of the cartilaginous elements (Fig.1F). Thus, for all subsequent experiments, we used 4 ng of MO egr1 spl. We conclude that Egr1 is required for pharyngeal cartilage development in zebrafish.

### Egr1 Regulates Late Chondrogenesis in Pharyngeal Skeleton

**Figure 2:**
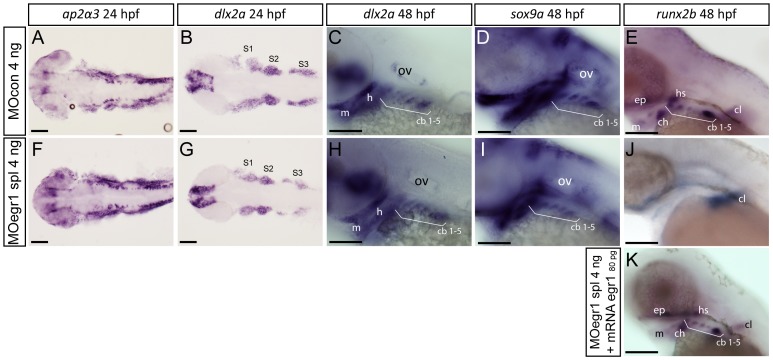
Only late chondrogenic and osteogenic marker genes display decreased expression in *egr1* morphants between 24 and 48 hpf. *In situ* hybridization was performed at the indicated stages for various cartilage markers, lateral views, anterior to the left. Scale bars 100 µm. (A–E) 4 ng MOcon treated control embryos, (F,G,H,I,J) 4ng splicing MOegr1 injected embryos and (K) rescue. (A,F) At 24 hpf, *ap2α3* expression in cranial neural crest cells (cNCC) is not altered in morphants. (B,C,G,H) cNCC marker *dlx2a* is normally expressed in e*gr1* morphants (G,H) compared to control embryos (B,C) at 24 and 48 hpf. (D,I) Expression of the essential chondrogenic gene *sox9a* is not changed at 48 hpf by *egr1* knock-down. (E,J,K) At 48 hpf, *runx2b* transcripts are absent in pharyngeal cartilage precursor cells in 4 ng MOegr1 spl embryos. Expression of *runx2b* is maintained in the cleithrum (cl) and ethmoid plate (ep). (K) Rescue by injection of 80 pg mRNA *egr1* restores all *runx2b* expression domains at 48 hpf. Otic vesicle (ov), mandible (m), ceratohyal (ch), hyosymplectic (hs), ceratobranchial pairs 1 to 5 (cb1-5), cleithrum (cl), ethmoid plate (ep), stream of cNCCs (S1–S3).

To specify the function of Egr1 during cartilage development, we assayed the expression of neural crest cell and cartilage markers in *egr1* morphant embryos by *in situ* hybridization. Expression of early neural crest markers *ap2a3* was not affected in *egr1* morphants at 24 hpf (Fig.2A,F). In addition, the expression of dlx2a, expressed in a subset of pre- and post-migratory cNCC that give rise to pharyngeal cartilage [52], [53] was not affected at 24 and 48 hpf in *egr1* morphants (Fig.2B,C,G,H). This indicates that Egr1 is not required for formation of cNCC or their migration into pharyngeal arches.

Proper chondrocyte stacking in cartilage requires the expression of Sox9a in these cells [6]. In *egr1* knock-down embryos, *sox9a* expression in pharyngeal chondrocytes remained unaffected at 48 hpf compared to control embryos (Fig.2D,I), indicating that the initial steps of chondrocyte differentiation are not affected following *egr1* knock-down.

Runx2b is a transcription factor known to play a key role in chondrocyte maturation and is expressed in mesenchymal cartilage condensations in the viscerocranium starting at 40 hpf [54], [55] and, at later stages in endochondral and intramembranous bone elements. In contrast to the earlier marker *sox9a*, expression of *runx2b* was abolished in 40 hpf (247/263, 93%) and 48 hpf (621/647, 95%) *egr1* morphants (Fig.2E,J). At 48 hpf, no expression was detected in the mandible, the hyoid nor in the ceratobranchials, a weaker expression was observed in the ethmoid plate while interestingly the expression remained normal in the cleithrum, an intramembranous bone. The specificity of this effect was confirmed by co-injection of 80 pg of *egr1* mRNA to 4 ng of MO egr1 spl (Fig.2K), revealing that exogenous Egr1 was able to rescue *runx2b* expression in the head cartilage (265/297, 89%).

We conclude that *egr1* expression is dispensable for early specification and migration of cNCCs, but is required for proper late chondrogenesis in pharyngeal arches.

### Egr1 is Expressed in Pharyngeal Endoderm and Oral Epithelium

**Figure 3:**
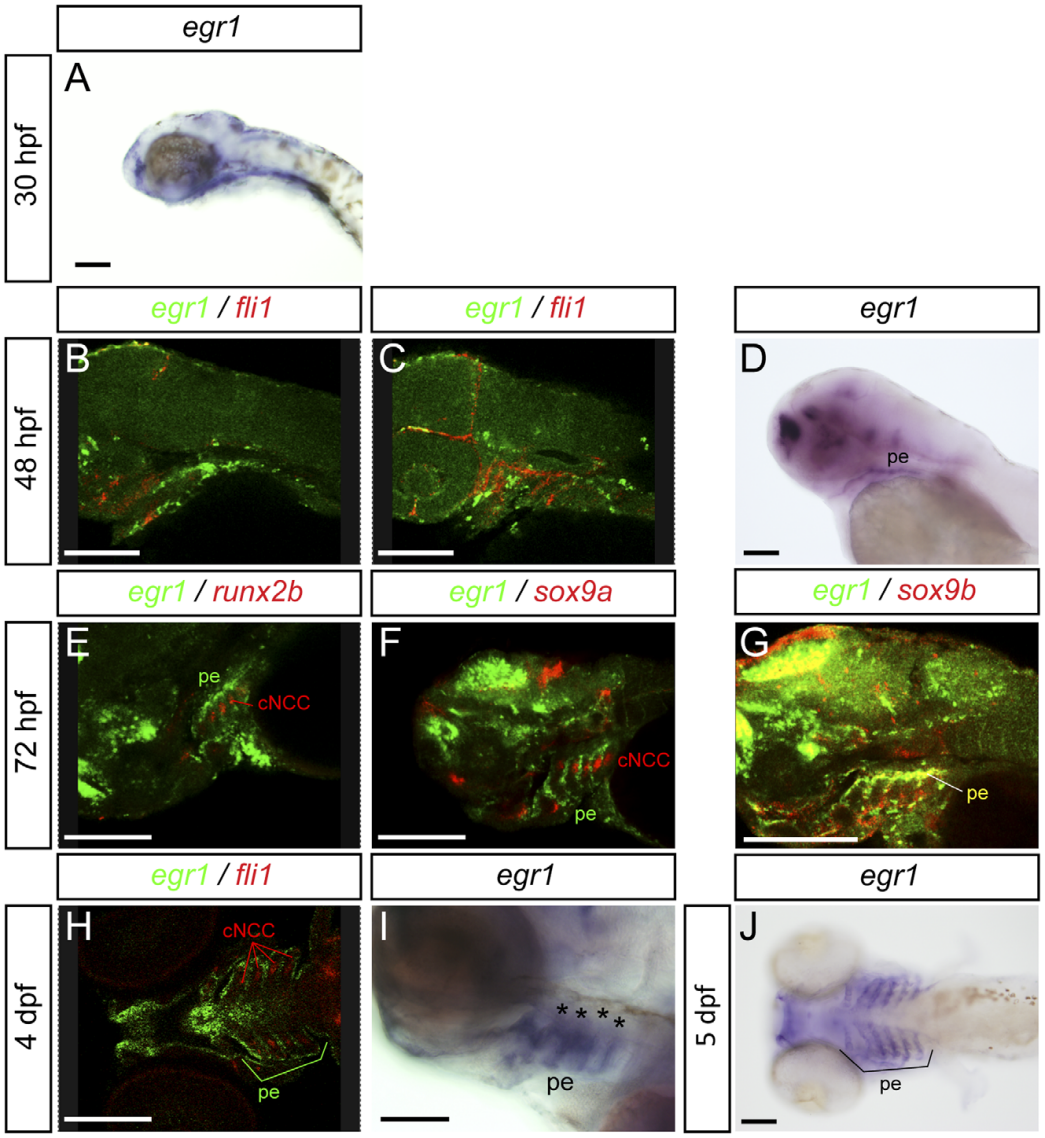
Expression of *egr1* in the pharyngeal region between 30 hpf to 5 dpf is restricted to endoderm and epithelium. Lateral (A–G,I) and ventral (H,J) views, anterior to the left. Scale bars 100 µm. Images of double *in situ* hybridizations were taken by confocal microscopy and pictures of individual Z-sections are shown. (A) e*gr1* transcripts are observed in the pharyngeal region starting at 30 hpf in endoderm. (B,C) At 48 hpf, double *in situ* hybridization for *egr1* (green) and *fli1* (red); *egr1* transcripts are localized in pharyngeal endoderm and do not colocalize with *fli1* mRNA in pharyngeal cartilage precursor cells. (D) *egr1* is expressed in pharyngeal endoderm. (E–G) At 3 dpf, e*gr1* (green) does not colocalize with *runx2b* (red) (E) or s*ox9a* (red) (F) in cartilage, while (G) e*gr1* (green) mRNAs colocalize with those for the pharyngeal endoderm marker *sox9b* (red). (H) At 4 dpf, e*gr1* (green) is never expressed in cells in pharyngeal cartilage precursor cells expressing *fli1* (red). (I) Expression of *egr1* at 4 dpf in pharyngeal endoderm. (J) At 5 dpf, e*gr1* is still expressed in pharyngeal endoderm (stars) and not in pharyngeal cartilage. Pharyngeal endoderm (pe), cranial neural crest cells (cNCC).

To better understand the cartilage defects observed in the absence of Egr1, we sought to determine the *egr1* expression pattern in the developing pharyngeal region. Whole-mount *in situ* hybridization against *egr1* confirmed *its* expression in the pharyngeal region starting at 30 hpf and persisting until at least 48 hpf (Fig.3A,D and [25]). At 48 hpf, *egr1* mRNA was detected in oral epithelium (Fig.3D) and in the endodermal pouches of the arches. This expression is observed until at least five days of development (Fig.3I,J). Expression in pharyngeal cartilage condensations was never observed.

To determine precisely in which tissue *egr1* is expressed, we performed double fluorescent *in situ* hybridizations at different stages of embryonic development using various markers for specific tissues. At 48 hpf, we performed a double hybridization for e*gr1* (in FITC, green) and *fli1* (in Cy3, red), which is expressed in pre-cartilage condensations and endothelium [2]. In confocal microscopy, the most lateral optical (longitudinal) sections reveal expression of *fli1* in the cartilage condensations while e*gr1* expression is located in stripes separating cartilages (Fig.3B, Movie S1). In more central sections, *egr1* mRNA is seen in the medial pharyngeal endoderm (Fig.3C, Movie S1). At three days of development, by comparing the expression of *runx2b* (Fig.3E), *dlx2a* (not shown) and *sox9a* (Fig. 3F, Movie S2) in the pharyngeal cartilage condensations with that of e*gr1*, we did not observe any colocalization. We can clearly see that at this stage e*gr1* is expressed in the pharyngeal endoderm and pouches that surround the pharyngeal cartilage. Furthermore, we observed co-expression of *egr1* with that of the endodermal marker s*ox9b* at three days in pharyngeal endoderm and pouches (Fig. 3G, Movie S3). At 4 dpf, the *egr1* expression domain is clearly surrounding the *fli1* domain in cartilage (Fig.3H, Movie S4). No colocalization was observed between e*gr1* and *fli1* at any stage. To confirm the expression of egr1 in the pharyngeal endoderm, we also carried out in-situ hybridization for *egr1* in casanova (*cas*) mutant embryos, devoid of all molecular endodermal markers [17]. In wild-type siblings (Fig. S1A,C), *egr1* transcripts were observed at 48 hpf in the pharyngeal region as well as in different brain regions and in the heart. In contrast, in 48 hpf homozygous *cas* embryos (Fig. S1B,D), no *egr1* expression was observed in the pharyngeal region (126/126, 100%), while expression is maintained in the brain, the duplicated hearts and increased in the fin buds.

We conclude that *egr1* is expressed in the pharyngeal endoderm of developing zebrafish embryos.

### Egr1 is Required for Pharyngeal Endoderm Expression of sox9b

The *casanova* mutant, devoid of endodermal tissue and known to be deficient in cartilage formation, [46] fails to express *runx2b* at 48 hpf ([46] and data not shown) similar to *egr1* morphants. Given the importance of pharyngeal endoderm for craniofacial cartilage development, we wished to test the role of Egr1 in this tissue.

**Figure 4:**
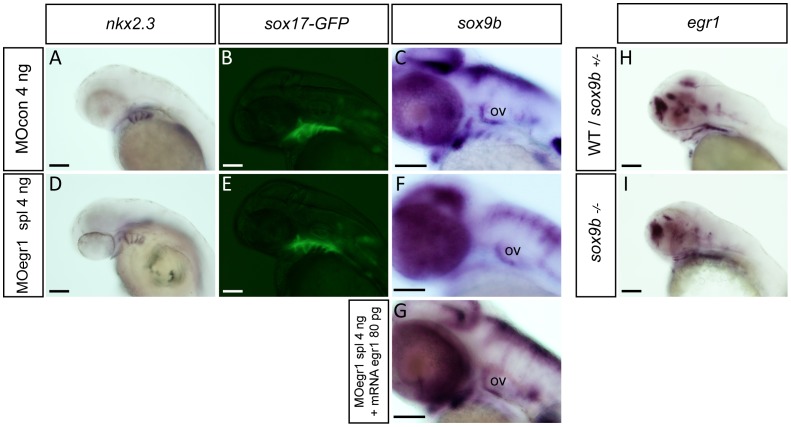
Egr1 is required for expression of *sox9b* in pharyngeal endoderm. Endodermal gene expression by *in situ* hybridization (A,C,D,F,G,H,I) or in living transgenic embryos (B,E) in control embryos (A–C,H), e*gr1* morphants (D–F), rescued embryos (G) and *sox9b* mutants (I) at 48 hpf. Lateral views, anterior to the left. Scale bars 100 µm. (A,D) *nkx2.3* expression is not altered in 4 ng MOegr1 spl injected embryos. (B,E) In living *sox17:GFP* transgenic embryos, the transgene is correctly expressed in *egr1* morphants. (D,F,G) The endodermal marker s*ox9b* is not expressed in the pharyngeal endoderm in 4 ng MOegr1 spl injected embryos, but its expression is rescued upon co-injection of 80 pg e*gr1* mRNA and spl 4 ng MOegr1. (H, I) In homozygous *sox9b^−/−^* embryos, *egr1* transcripts are still observed in the pharyngeal endoderm like in the wild-type or heterozygous *sox9b^+/−^* embryos. Pharyngeal endoderm (pe), otic vesicle (ov).

To determine whether Egr1 is required for endoderm formation, we tested whether pharyngeal endoderm is still present in *egr1* morphants. By *in situ* hybridization, expression of the endodermal marker *nkx2.3* was still present in e*gr1* morphants at 48 hpf, although some alterations in the precise shape of the pouches are observed relative to control embryos, probably reflecting the described defects in cNCC cells (Fig.4A,D). Similarly, *sox17-GFP* transgenic embryos expressing GFP in the entire endoderm, previously injected with MOegr1 spl morpholino display a normal pharyngeal GFP expression until 72 hpf (Fig.4B,E). Thus, Egr1 depletion does not prevent formation of the pharyngeal endoderm.

At stages beyond 26 hpf, expression of the Sox9b transcription factor is localized in pharyngeal epithelium and endoderm; this factor indirectly regulates *runx2b* expression in the neighboring perichondrium and chondrocytes and controls chondrocyte proliferation, cell death and patterning [5]. In homozygous *sox9b* ^b971^ mutants, cranial *runx2b* expression is only maintained in the cleithrum (84/88, 95%), reminiscent of the situation observed in *egr1* morphants.

Knowing that e*gr1* is expressed in pharyngeal endoderm, we investigated potential regulatory connections between the *sox9b* and *egr1* genes. At 42 hpf, *egr1* morphants do not express *sox9b* in the pharyngeal pouches and its expression in the brain is altered (156/171, 91%) (not shown). At 48 hpf, *sox9b* mRNA is still absent in the branchial arches of e*gr1* morphants, while a decreased expression relative to controls is observed in the two first pharyngeal arches (432/462, 93%) (Fig.4C,F). The observed decreased *sox9b* expression was rescued by injecting 80 pg of e*gr1* mRNA along with 4 ng of *egr1* splicing morpholino, showing its specificity for Egr1 knock-down (264/311, 84%) (Fig.4G). Conversely, in *sox9b* mutant (*sox9b^b971^*) embryos, the e*gr1* expression pattern remains intact in the pharyngeal endoderm and epithelium as compared to wild type control siblings (Fig.4H,I). Our results demonstrate that Egr1 is required for *sox9b* expression in pharyngeal endoderm.

### Runx3 Controls Cartilage Development by Regulating *egr1* and *sox9b* Expression in Pharyngeal Endoderm

In zebrafish, *runx3* is another gene expressed in pharyngeal endoderm and required for *runx2b* expression in the ventral pharyngeal chondrocytes [46], similar to the *egr1* and *sox9b* genes. Therefore, we decided to investigate the contribution of the *runx3* gene to the regulatory cascade in pharyngeal endoderm by performing epistasis experiments.

**Figure 5:**
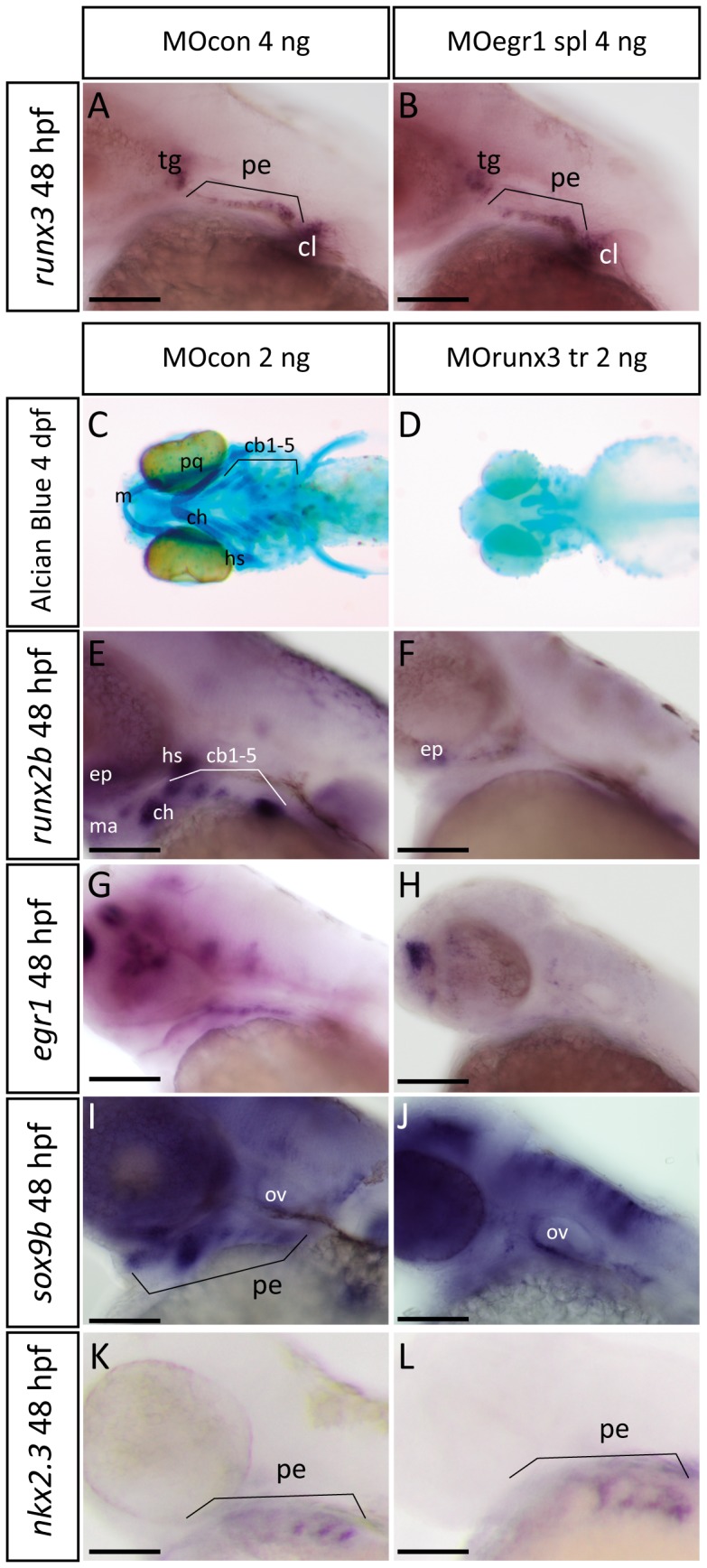
Runx3 is required for pharyngeal *egr1* and *sox9b* expression at 48 hpf. Lateral views of *in situ* hybridizations (A,B,E–L) with indicated markers and ventral views of Alcian Blue stained embryos (C,D), anterior to the left. Scale bars 100 µm. (A,B) Endodermal *runx3* expression in the pharyngeal region is not altered in 4 ng MOegr1 spl morphants. (C,D) *runx3* knock-down using 2 ng MOrunx3 tr leads to total absence of viscerocranium and the anterior neurocranium (D) compared to control (C) embryos. (E,F) *runx3* morphants do not express r*unx2b* in pharyngeal cartilage precursor cells. (G,H) *runx3* morphants do not express e*gr1* transcripts in pharyngeal endoderm. (I,J) The endodermal marker s*ox9b* is absent in pharyngeal endoderm when *runx3* expression is blocked. (K,L) *runx3* knock-down does not affect expression of pharyngeal endodermal marker *nkx2.3 a*t 48 hpf. Trigeminal ganglia (tg), pharyngeal endoderm (pe), cleithrum (cl), Meckel’s cartilage (m), palatoquadrate (pq), hyosymplectic (hs), ceratohyal (hs), ceratobranchials 1 to 5 (cb1-5), ethmoid plate (ep), otic vesivle (ov).

By *in situ* hybridization, we showed that *runx3* expression at 48 hpf was similar in endodermal pouches of *egr1* morphants (102/106, 96%) to that in control embryos (Fig.5A,B). Conversely, to investigate the function of Runx3 in e*gr1* and consequently also s*ox9b* and *runx2b* expression, we performed *runx3* depletion using well established morpholino-mediated knock-down [46]. As expected, injection of 2 ng of *runx3* morpholino into wild-type eggs resulted in a complete absence of viscerocranium and the anterior part of the neurocranium, as revealed by Alcian Blue cartilage staining at 4 dpf (123/137, 92%) (Fig.5C,D). We also confirmed that *runx3* knock-down disrupts *runx2b* expression in pharyngeal arch mesenchyme and that only a small expression remains in basicranial anlagen at 48 hpf (251/273, 91%) (Fig.5E,F). Importantly, *runx3* morpholino injected embryos completely lost e*gr1* transcripts in the pharyngeal region, while these were maintained in the telencephalon at 48 hpf compared to control morpholino-injected siblings (310/347, 89%) (Fig.5G,H). Consistent with our previous observations, *sox9b* expression in pharyngeal endoderm was also disrupted at 48 hpf in *runx3* morphants (172/198, 86%) (Fig.5I,J). Finally, *nkx2.3* expression was maintained (Fig.5K,L) indicating that the endoderm itself was present.

**Figure 6:**
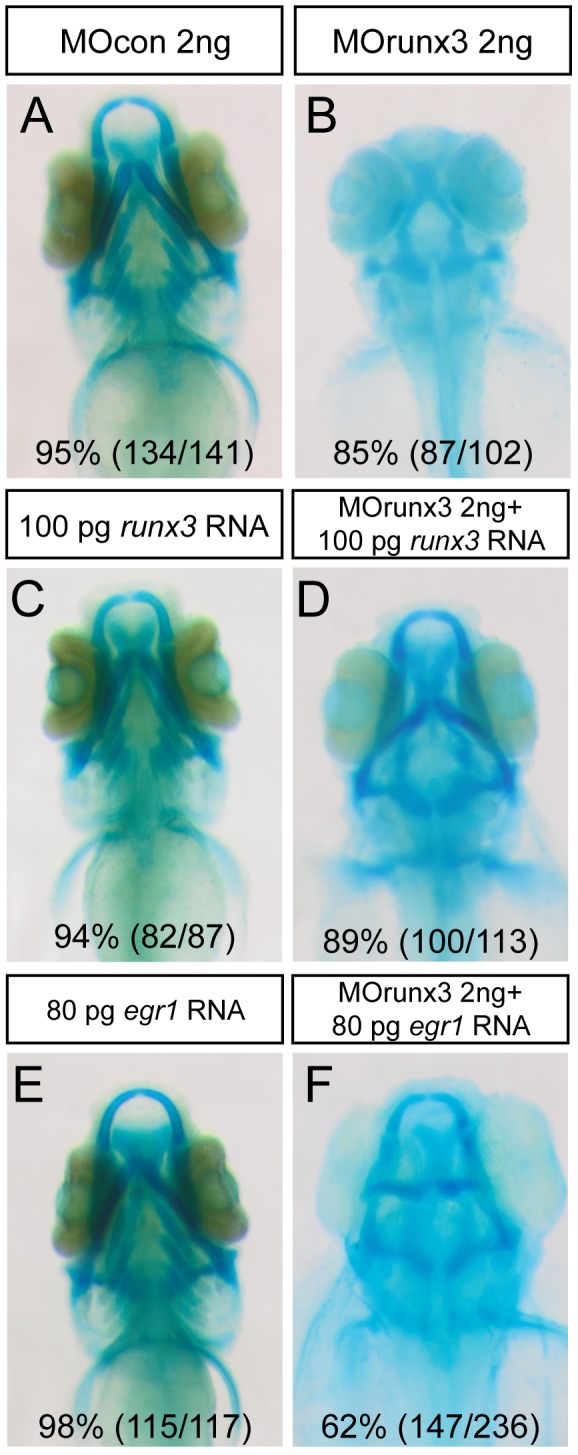
Runx3 depleted embryos can be rescued by *runx3* and *egr1* mRNA. (A–F) Head cartilages were stained with Alcian Blue in morpholino treated larvae at 4 dpf; ventral views are shown. (A) MOcon 2 ng injected larvae. (B) MOrunx3 2 ng injected larvae do not develop viscerocranium. (C) 100 pg of *runx3* mRNA do not affect 4 dpf old larvae cartilage morphology. (D) Injection of 100 pg *runx3* mRNA rescues 89% of MOrunx3 2 ng injected eggs. (E) 80 pg of *egr1* mRNA. (F) Injection of 80 pg *egr1* mRNA rescues 62% of MOrunx3 2 ng injected eggs.

When we tested the specificity of the effects observed in *runx3* morphants by alcian blue staining at 4 dpf, we observed that co-injection of*runx3* mRNA caused a partial rescue in 89% of the larvae ( Fig.6A,B,D ), while *runx3* mRNA alone had no effect ( Fig.6C ). As shown before, injection of *egr1* mRNA had no effect ( Fig.6E ), however its co-injection with MO runx3 caused a partial rescue of the anterior part of the viscerocranium in 62% of the larvae ( Fig.6F ), indicating that Runx3 is indeed acting through activation of *egr1* expression.

In conclusion, our results show that in pharyngeal endoderm Runx3 is required for *egr1* expression, which in turn is required for s*ox9b* expression and, finally Sox9b presumably triggers an extracellular signal leading to *runx2b* expression in post-migratory cNCC and chondrogenesis.

### Egr1 Down Regulates Follistatin a Expression in Pharyngeal Endoderm and Cartilage

To determine which extracellular signal is controlled by the endodermal regulatory cascade, we decided to analyze different candidate ligands of the HH and BMP signaling pathways in *egr1* morphants. *Shh* is expressed in pharyngeal endoderm [15], its expression is not affected in e*gr1* morphants compared to control embryos at 48 hpf. Among the BMP factor family, *bmp2a*, *bmp2b*, *bmp4*, *bmp5* and *bmp7* are all expressed in pharyngeal endoderm. By *in situ* hybridization in Egr1 depleted embryos, no significant alteration of expression was observed in the pharyngeal region for any of these genes (data not shown).

The gene coding for the secreted TGFß/BMP antagonist follistatin A (Fsta) is expressed in presumptive cephalic mesendoderm at 8 hpf [47], [56] and at later stages in arch vasculature and skeleton [57], [58]. In homozygous *casanova* mutants, devoid of endoderm, *sox9b* mRNA was undetectable (92/96, 95%) (Fig. S2A,B) and *fsta* expression is abolished (57/61, 93%) (Fig. S2C,D) in the branchial region at 48 hpf, confirming the expression of both genes in pharyngeal endoderm or at least the requirement of this tissue for their expression in this region.

**Figure 7:**
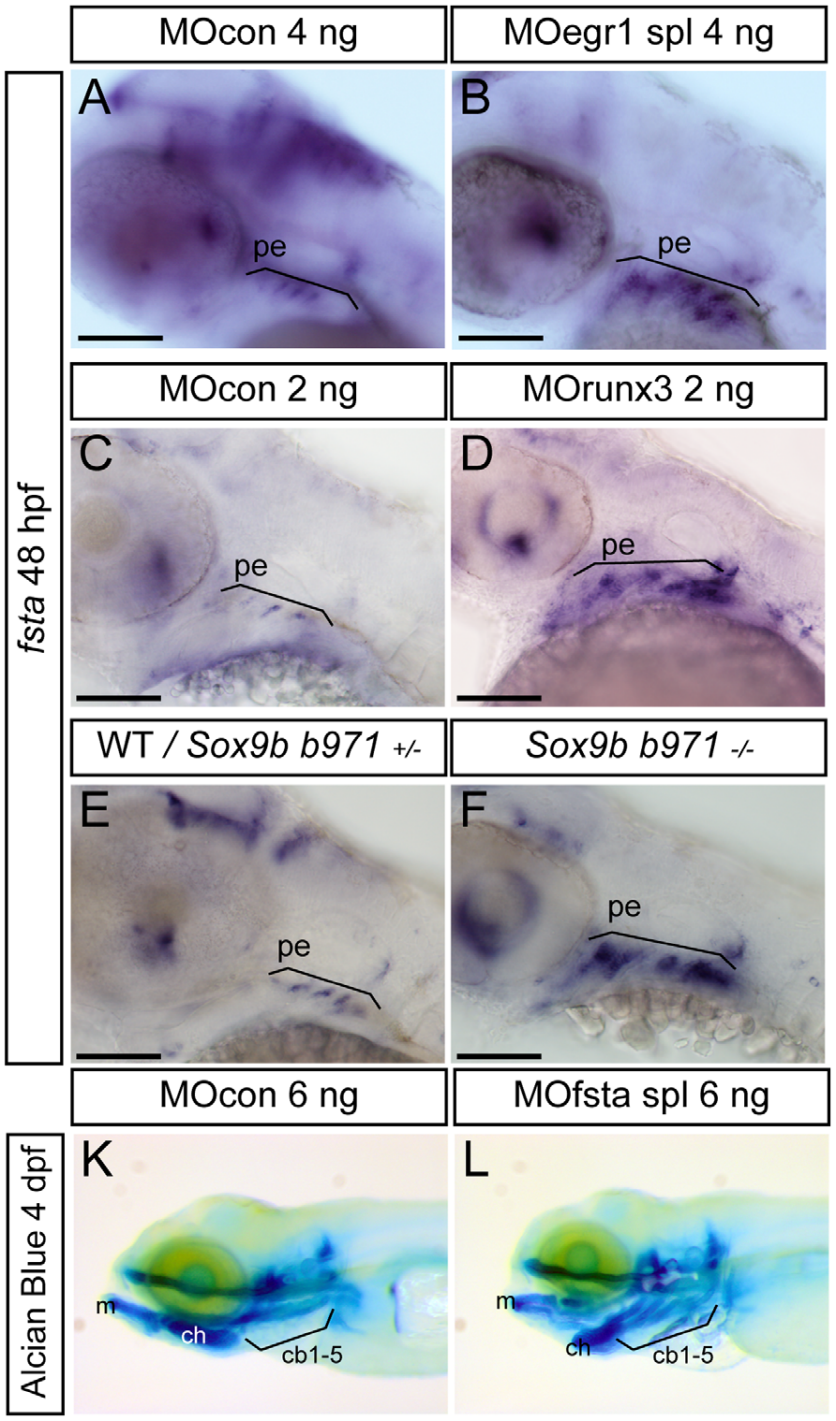
Expression of *fsta* is increased in *runx3* and *egr1* morphants and *sox9b* mutants. Lateral views of *in situ* hybridizations, anterior to the left. Scale bars 100 µm. (A–F) Compared to controls or wild-type embryos, expression of *fsta* is up-regulated in e*gr1* morphants (A,B), *runx3* morphants (C,D), and homozygous *sox9b* mutants (E,F) at 48 hpf. pharyngeal endoderm (pe). (G,H) 4 dpf Alcian Blue stained larvae injected with MOcon 6 ng (K) and MOfsta 6ng (L). Knock-down of *fsta* causes a hyperplasia of the viscerocranium. Meckel’s cartilage (m), ceratohyal (ch), ceratobranchials 1 to 5 (cb1-5).

In *egr1* morphants, a clear over-expression of *fsta* (274/302, 90%) relative to controls was observed at two days of development (Fig.7A, B). The *fsta* expression domain is more intense and extended in MOegr1-injected relative to MOcon-injected embryos, indicating that Egr1 is required for inhibition of *fsta* expression in wild type embryos. Similarly, embryos depleted of Runx3 by microinjection of MOrunx3 (157/170, 92%) (Fig.7C,D), or of Sox9b in the homozygous *sox9b^b971I^* mutant (77/79, 97%) (Fig.7E,F) displayed a dramatic increase of *fsta* expression and an extension of its expression domain as compared to wild type embryos. Thus, depletion of any of the endodermal transcription factors leads to increased *fsta* expression.

To further characterize a putative function of Follistatin A in cartilage development, we decreased its expression by injecting a splicing morpholino against *fsta* [47]. Injection of 6 ng of MOfsta spl resulted in 85% (86/101) of 4 dpf larvae displaying clearly increased cartilage elements compared to the controls (Fig.7G,H), showing that Follistatin A indeed plays an inhibitory role on the formation of head cartilage.

Taken together, these results indicate that the endodermal cascade of transcription factors Runx3, Egr1 and Sox9b is required to reduce the level of fsta expression in the pharyngeal region.

### BMP Signaling is Required for Pharyngeal Cartilage Formation and *runx2b* Expression in Cartilage

**Figure 8:**
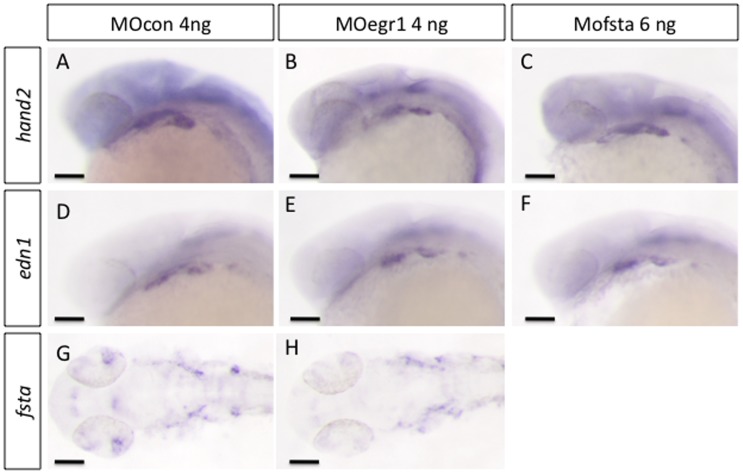
*egr1* and *fsta* knock-down do not affect ventralisation of cranial neural crest cells. *In situ* hybridization was performed at 24 hpf, lateral views, anterior to the left. Scale bars 100 µm. (A,D,G) MOcon 4 ng, (B,E,H) MOegr1 4 ng and (C,F) MOfsta 6 ng. No modification in the expression of markers *hand2*, *edn1* and *fsta* was observed in MOegr1 4 ng or MOfsta 6 ng injected embryos compared to control.

In vertebrates, the BMP pathway is known to play an essential role in skeletogenesis [12], but also many early developmental processes such as gastrulation or neurulation [59]. In particular, BMP signaling directs ventral patterning of the viscerocranium before 24 hpf [14]. Most of the factors investigated here are expressed in the pharyngeal region at stages beyond 24 hpf and the defects caused by their depletion are also observed at these later stages. To investigate whether Egr1 or FstA might be required for early ventral patterning of the pharyngeal arches, we tested the expression of the ventral markers *hand2* and *edn1* at 24 hpf in *egr1* and *fsta* morphants. Expression of both ventral markers was maintained in the microinjected embryos (Fig.8A–F), indicating that dorso-ventral patterning is not affected by depletion of Egr1 or FstA. We also showed that the weak *fsta* expression at 24 hpf is not affected in *egr1* morphants (Fig.8G,H), further supporting the notion that the endodermal regulatory cascade acts beyond 24 hpf. Similar results were obtained at 30 hpf (data not shown).

**Figure 9:**
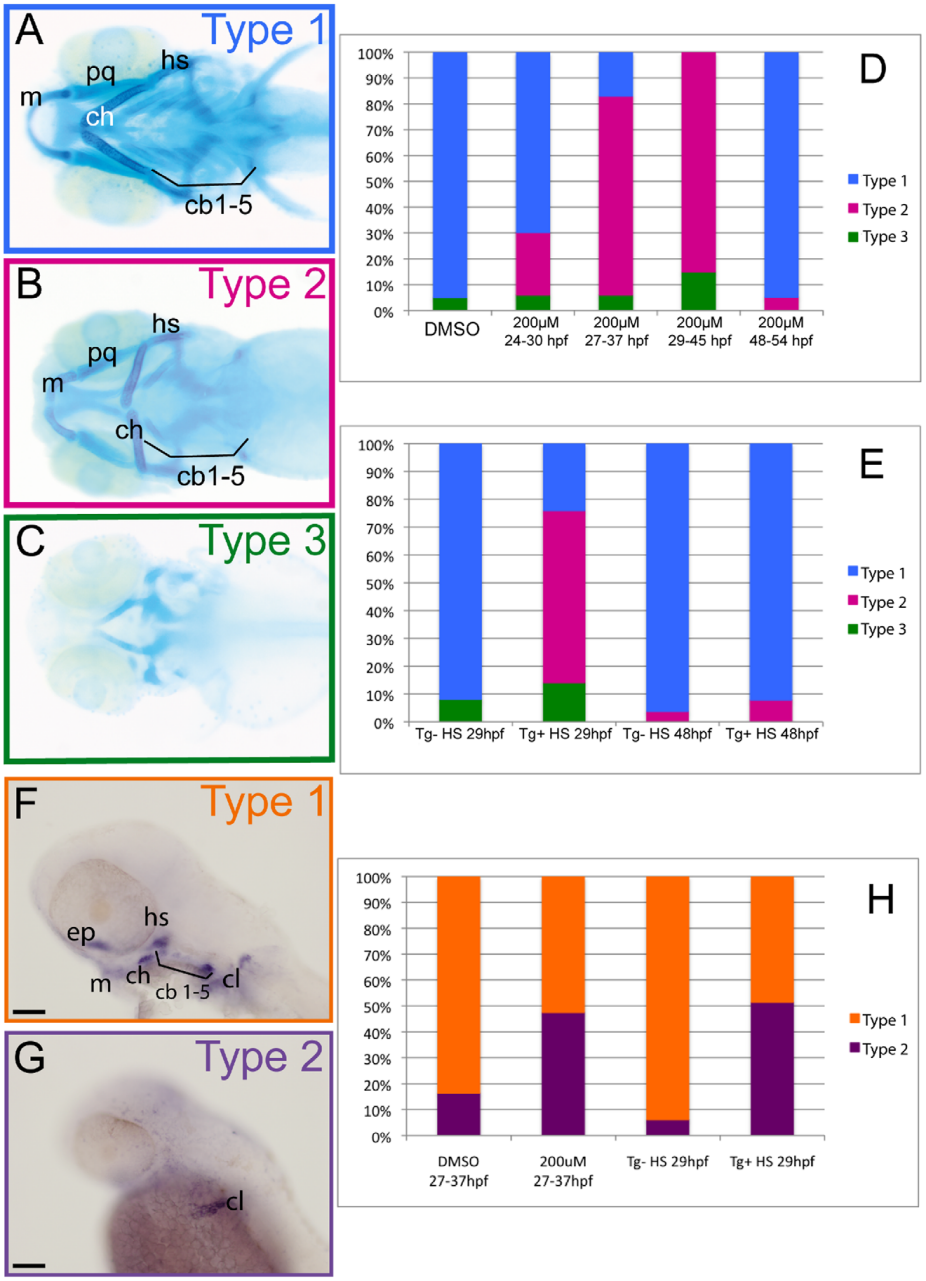
BMP signaling is required between 27 and 37 hpf for *runx2b* expression and head cartilage development. (A–E) Cartilage was stained with Alcian Blue in 4 dpf larvae, ventral views are shown, anterior to the left. (A) Type 1 larvae (blue) display a wild-type morphology, all cartilage elements are present and well shapped. (B) Type 2 larvae (pink) lack ceratobranchials and have mis-shaped Meckel’s cartilage, palatoquadrate, ceratohyal and hyosymplectic. (C) Type 3 larvae (green) display a complete absence of viscerocranium and a reduction of the anterior neurocranium. (D,E) Graphs representing the proportions of the three types of larvae after the indicated treatments. (D) Treatment with the BMP inhibitor dorsomorphin (200 µM) most severely affects head cartilage between 29 and 45 hpf; (DMSO) dimethylsulfoxide. (E) Heat shock treatment of *(hsp70l:dnBmpr-GFP)w30* transgenic embryos between 27 hpf and 37 hpf most severely affects pharyngeal cartilage development. (F–H) *In situ* hybridization for *runx2b* expression at 48 hpf, lateral views, anterior to the left, scale bars 100 µm. (F) Type 1 embryos (orange) have a normal *runx2b* expression pattern in all pharyngeal cartilage precursor cells, cleithrum and ethmoid plate. (G) Type 2 embryos (purple), compared to type 1 embryos, only express *runx2b* in cleithrum and weakly in the ethmoid plate. (H) Graph representing the proportions of the two types of larvae after the indicated treatments. Dorsomorphin treatment of wt embryos and heat shock treatment of *(hsp70l:dnBmpr-GFP)w30* between 27 hpf and 37 hpf decreases *runx2b* expression in pharyngeal cartilage, but not in the cleithrum. (DMSO) dimethylsulfoxide, (Tg+) Transgene expressing embryo, (Tg-) Transgene non-expressing siblings, (HS) heat shock. Meckel’s cartilage (m), palatoquadrate (pq), ceratohyal (ch) and hyosymplectic (hs), ceratobranchials 1 to 5 (cb1-5).

To assess the role of BMP signaling in head cartilage formation at later stages of development without affecting earlier processes, we investigated the effects of dorsomorphin, a selective inhibitor of ALK2, BMPR-IA and BMPR-IB signaling and of BMP-induced Smad1/5/8 phosphorylation [60] at different stages beyond 24 hpf. The effects on cranial cartilage formation were analyzed by Alcian blue staining at 4 dpf and the treated larvae were classified according to the extent of the defects seen in cartilage formation (Fig.9A–C). Type 1 was considered as wild-type cartilage morphology, type 2 larvae displayed a severe reduction or absence of the five pairs of branchial arches and a malformation and reduction of the first two pairs of pharyngeal arches (mandible and hyoid). Finally, type 3 larvae are characterized by a complete absence of pharyngeal arches (viscerocranium) and severe reduction of the neurocranium.

Treatment of embryos with 200 µM dorsomorphin between 24 and 30 hpf resulted in 70% (138/197) of type 1 larvae, 23% (47/197) of type 2 larvae and 6% (12/197) of larvae with a complete loss of viscerocanium (type 3), while after treatment between 27 hpf and 37 hpf, we observed only 6% (14/231) of type 1 larvae, 77% (177/231) of type 2 larvae and finally 17% (40/231) of type 3 larvae (Fig.9D). Treatment between 29 hpf and 45 hpf resulted in 85% (218/257) of type 2 larvae and 15% (39/257) of type 3 larvae. In contrast, dorsomorphin treatment performed between 48 hpf and 54 hpf led to 88% (174/198) of the drug treated larvae displaying a type 1 cartilage morphology and only 12% (24/198) with a type 2 cartilage morphology. These results clearly confirm that the BMP pathway is required for proper cartilage formation in zebrafish at these later stages, comparison of the different experiments reveals that the most crucial period lies between 27 hpf and 37 hpf.

To confirm this requirement for BMP signaling at late stages, we also used an inducible dominant negative BMP receptor-GFP transgenic line (*hsp70l:dnBmpr-GFP)w30*. We performed a heat shock at 37°C during 30 minutes at 29 hpf and compared the head cartilage at 4 dpf of the transgenic larvae to that of their non-transgenic siblings (Fig.9E). While 92% (140/152) of control larvae formed a perfectly normal type 1 cartilage, indicating that the heat shock itself had no effect, the transgenic larvae (positive for GFP expression) producing the active dominant negative BMP receptor presented a clear alteration of chondrogenesis, with 62% (106/171) belonging to type 2 cartilage morphology, 24% (41/171) type 1 cartilage morphology and 14% (147/171) type 3 cartilage morphology. When heat shock on *(hsp70l:dnBmpr-GFP)w30* was performed after 48 hpf, almost all larvae presented normal cartilage (123/129, 95%) (type 1). These results clearly confirm that BMP signaling is required for pharyngeal cartilage formation between 27 hpf and 37 hpf.

The cartilage defects observed upon BMP inhibition are quite similar to those obtained in e*gr1* and *runx3* morphants or *sox9b* mutants. Therefore, we also analyzed *runx2b* expression at 48 hpf in embryos treated with 200 µM dorsomorphin between 27 hpf and 37 hpf (Fig.9F–H). Half of the treated embryos did not express *runx2b* in pharyngeal arches (285/543, 52%), while it remained expressed in the ethmoid plate and cleithrum. Upon heat shock of (*hsp70l:dnBmpr-GF*)*w30* transgenic animals at 29 hpf, we observed the same percentage (33/66, 50%) of embryos expressing *runx2b* only in the cleithrum at 48 hpf.

Taken together, these observations indicate that inhibition of BMP signaling between 29 and 37 hpf causes cartilage defects at 4 dpf and prevents *runx2b e*xpression at 2 dpf, similar to the defects observed upon blocking of the Runx3-Egr1-Sox9b cascade in the endoderm.

### Egr1 is Required for BMP Signaling in the Pharyngeal Region

**Figure 10:**
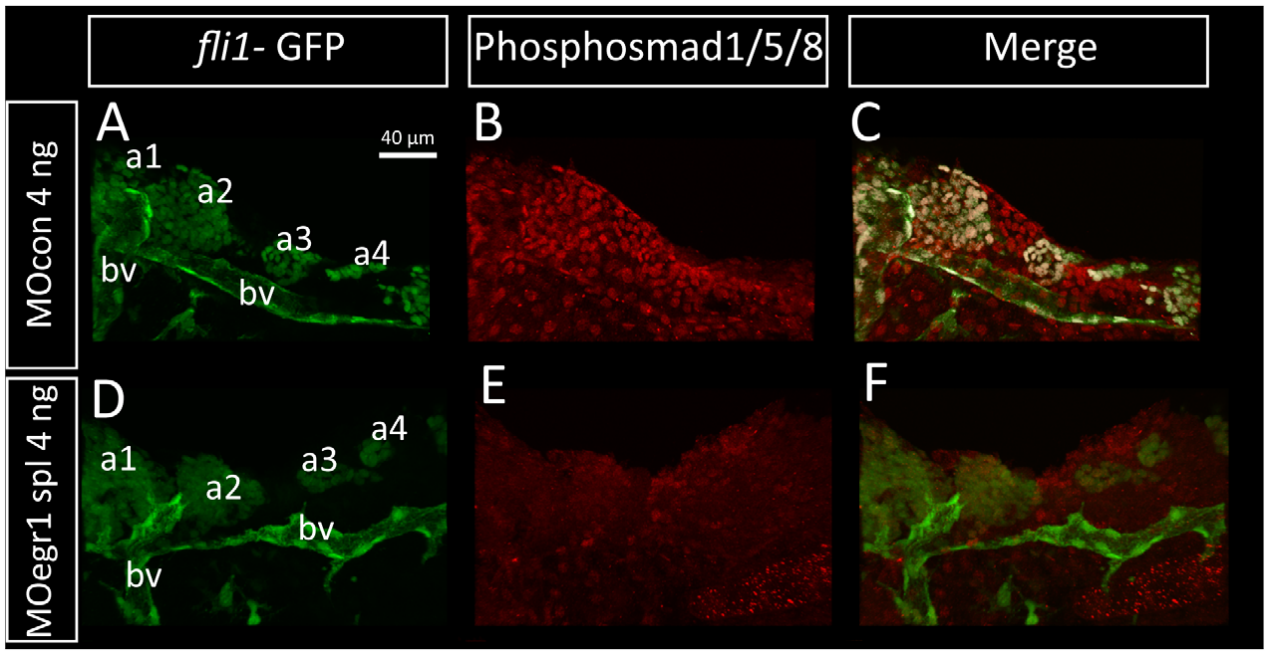
Bmp signaling is down-regulated in *egr1* morphants. Pharyngeal cartilage precursor cells were visualized by immunohistochemistry using anti-GFP antibodies (green) in *fli-*GFP embryos. Activity of the BMP signaling pathway was assessed using antibodies against phospho-Smad1/5/8 (red) in 32 hpf embryos. Ventral view of pharyngeal arches, scale bar 40 µm. (A–F) Pharyngeal cartilage precursor cells were visualized by immunohistochemistry using anti-GFP antibodies (green) in *fli1-*GFP embryos. Activity of the BMP signaling pathway was assessed using antibodies against phospho-Smad1/5/8 (red) in 32 hpf embryos. Ventral view of pharyngeal arches, scale bar 40 µm. (A,B,C) 4 ng MOcon injected embryos, (D, E, F) 4 ng MOegr1 spl injected embryos. *fli1-*GFP embryos express the GFP transgene in cartilage precursors and endothelial cells in control (A) and in e*gr1* morphants (D). In contrast, phospho-Smad1/5/8 is is clearly down regulated in e*gr1* morphants (E) compared to control embryos (B). (C,F) Overlay images of the two anti-body signals clearly show that phospho-Smad1/5/8 is present in GFP-epressing cartilage precursor cells in control embryos (C), while no colocalization is observed in e*gr1* morphants (F). (a1) first arch, (a2) second arch, (a3) third arch, (a4) fourth arch, (bv) blood vessel.

Finally, we determined whether the Runx3-Egr1-Sox9b cascade is required to maintain normal BMP signaling in cartilage precursors by reducing Fsta signaling in the pharyngeal arches. To this end, we performed double fluorescent immunohistochemistry using anti-phospho-Smad1/5/8 and anti-GFP antibodies on *fli1*-GFP embryos. We used *fli-GFP* expressing embryos in order to visualize the cNCC through the presence of GFP. The embryos were injected with a control morpholino or the *egr1* splicing morpholino and fixed at 32 hpf to perform immunohistochemistry (Fig.10A–F).

By confocal analysis, we detected the presence of phospho-Smad1/5/8 in the entire pharyngeal arch region including the *fli1*-expressing cranial cartilage precursor cells in 32 hpf old MOcon embryos, indicating that BMP signaling is active at 32 hpf in control embryos (Fig.10A–C). When we analyzed e*gr1* morphants, no phospho-Smad1/5/8 was detected in the pharyngeal region (Fig.10D–F). In addition, we could also confirm that *fli1*-GFP expressing cNCCs are disorganized in *egr1* morphants compared to controls. Altogether, our results demonstrate that Egr1 and the regulatory cascade Runx3/Egr1/Sox9b/Fsta expressed in the pharyngeal endoderm is absolutely required for BMP activation in cartilage around 29 hpf.

## Discussion

### Pharyngeal Endoderm Hosts a Regulatory Cascade of Transcription Factors Required for Formation of the Viscerocranium

In this study, we identified a regulatory cascade composed of three transcription factors: Runx3, Egr1 and Sox9b occurring beyond 24 hpf in zebrafish pharyngeal endoderm. This cascade is required for inhibition of *fsta* expression, coding for a known secreted BMP inhibitor, thereby allowing proper BMP signaling to the developing cNCCs.

We have shown that all three factors are coexpressed in pharyngeal endoderm starting at about 30 hpf and that each of them is absolutely required for pharyngeal cartilage formation. These results are in agreement with previous studies concerning the function of Sox9b and Runx3 in zebrafish. Expression of *runx3* was found in pharyngeal pouches at 2 dpf and in endodermally derived oral epithelium at 3 dpf [46]. Our own loss of function experiments confirmed that *runx3* morphants nearly completely lack head cartilage [46] and lost *runx2b* expression in pharyngeal cartilage. Similarly, *sox9b* is expressed (starting at 26 hpf) in pharyngeal endoderm and *sox9b* mutants or morphants display a dramatic reduction of pharyngeal cartilage at 4 dpf and a lack of *runx2b* expression at 48 hpf, while exogenously expressed Sox9b partially rescued the mutant phenotype [5]. Thus, at later stages endodermal Runx3 and Sox9b regulate cartilage and bone development by indirectly controlling r*unx2b* expression in cNCC cells.

Here, we introduce a new player by showing that the endodermal transcription factor Egr1 is required for cartilage formation and expression of s*ox9b* in endoderm and *runx2b* in cNCCs. Expression of *egr1* in endodermal pouches was deduced from single and double fluorescent *in situ* hybridization experiments, co-expression of *egr1* with cNCC markers such as *dlx2a* (not shown) or *sox9a* was never observed. The complete absence of *egr1* mRNA in the pharyngeal region of *cas* mutants, lacking endoderm, further supports this conclusion. The defects were observed following gene knock-down using translation or splicing morpholinos and the specificity of these defects for Egr1 depletion was shown by the rescue experiments.

Additional experiments revealed that Runx3 depletion led to decreased expression of both *egr1* and *sox9b*, while *runx3* expression was not affected in *egr1* morphants or *sox9b* mutants. Egr1 depletion decreases *sox9b* expression only, while conversely *egr1* expression is not affected in *sox9b* mutants. Finally, *sox9b* mutants display normal expression of *egr1* and *runx3*. We further show that the defects observed in *runx3* morphants can be partially rescued by expression of exogenous *runx3* or *egr1* mRNA, clearly indicating that Runx3 is located upstream of Egr1. Taken together, these results establish a regulatory cascade where Runx3 activates expression of Egr1, which itself then activates *sox9b* transcription. This cascade is not required for pharyngeal endoderm formation or the survival of pharyngeal endodermal cells, as was previously shown for Runx3 morphants [46]. We similarly confirmed that in *egr1* or *runx3* morphants or *sox9b* mutants, expression of the endodermal markers *nkx2.3* and *sox17* is not altered compared to controls. In conclusion, we describe a regulatory cascade that operates mainly in the pharyngeal endoderm and controls expression of *runx2b* in cartilage mesenchyme as well as cartilage differentiation and morphogenesis. This control exerted by endodermal transcription factors is obviously mediated by an extracellular signaling pathway that remains to be described.

### Endodermal Signaling Controls the BMP Pathway in Cartilage Precursor Cells

One of the signaling pathways involved in cartilage and bone formation is the TGFß/BMP pathway [12]. BMP ligands bind to their transmembrane receptor complex, consisting of a type I and a type II receptor, to induce phosphorylation of the type I receptor. The activated receptor (Alk1, 2, 3 or 6) then phosphorylates Smad1, 5 and/or 8 which in turn associate with their common partner, Smad4 to migrate into the nucleus and regulate target genes [61]. Craniofacial defects were reported in conditional knock-out mice lacking BMP type I receptor Alk2 [62] or Smad4 [63] in cNCC, or in transgenic mice expressing the antagonistic Smad7 in cNCC cells [64]. In zebrafish, several members of the BMP ligand family, such as Bmp2a, Bmp2b, Bmp4, Bmp5 and Bmp7 were shown to be secreted in the pharyngeal region [12], [65], [66] and their importance for head cartilage development was shown [67]. Recently, BMPs were shown to promote ventral fates of the craniofacial skeleton in zebrafish [14]. Based on our previous experiments, we therefore tested the involvement of the BMP pathway in cartilage formation by inhibiting BMP signaling at stages beyond 24 hpf. Treatment with the specific inhibitor dorsomorphin [60] revealed that the importance of BMP signaling for visceral cartilage formation and *runx2b* expression increases after 24 hpf and is most crucial during the period between 27–37 hpf. This result was confirmed by inducing the expression of a dominant-negative Bmp receptor [45] at 29 hpf. This period coincides with the time of onset of *runx3* [46], *egr1* and *sox9b* gene expression in pharyngeal endoderm.

When we tested the expression of various extracellular signaling molecules (Shh, Fgf3, Fgf8, Bmps) in *egr1* morphants, we did not detect any decrease of expression for any of these genes. During development, many processes require inhibition of BMP signaling by secreted BMP antagonists [56], [59], [68], [69]. Follistatin was first described as a polypeptide inhibiting the release of follicle stimulating hormone in the pituitary [70]. Since then, its involvement in ovarian development and function and its antagonism to members of the TGFß/BMP family has been extensively studied [56], [71], [72]. The function of Follistatin as BMP antagonist in skeletal development has also been described [56], [69], [73]. We observed a strongly increased expression of *follistatin a* (*fsta*) in the pharyngeal region at 48 hpf in *egr1* morphants. Such a strong over-expression of Follistatin A would obviously lead to a perturbation of BMP signaling in the entire pharyngeal region and cause defects similar to the BMP inhibition experiments discussed above. Indeed, when we tested activation of the BMP pathway using antibodies against phospho-Smad1/5/8, we observed that *egr1* knock-down dramatically reduces BMP signaling in the pharyngeal region. Pathway activation was abolished not only in the post-migratory cNCC clusters, but also in the neighboring tissues, potentially causing additional perturbations in skeletal morphogenesis.

Clearly, the function of BMP signaling in craniofacial development is conserved in vertebrates, including mouse and human, and the importance of antagonists such as Follistatin is also well documented. Different threshold levels of Bmp4 were shown in mouse to regulate various genes involved in craniofacial skeletal morphogenesis [74]. Conservation of the endodermal function of Sox9b is more difficult to assess due to the fact that the diverging functions of zebrafish Sox9a and Sox9b are covered by their single mammalian ortholog Sox9. It is however interesting to note that an increased expression of Follistatin was observed in male gonads of *Sox9* knock out mice [75], suggesting a possible repressive function similar to that described here.

**Figure 11:**
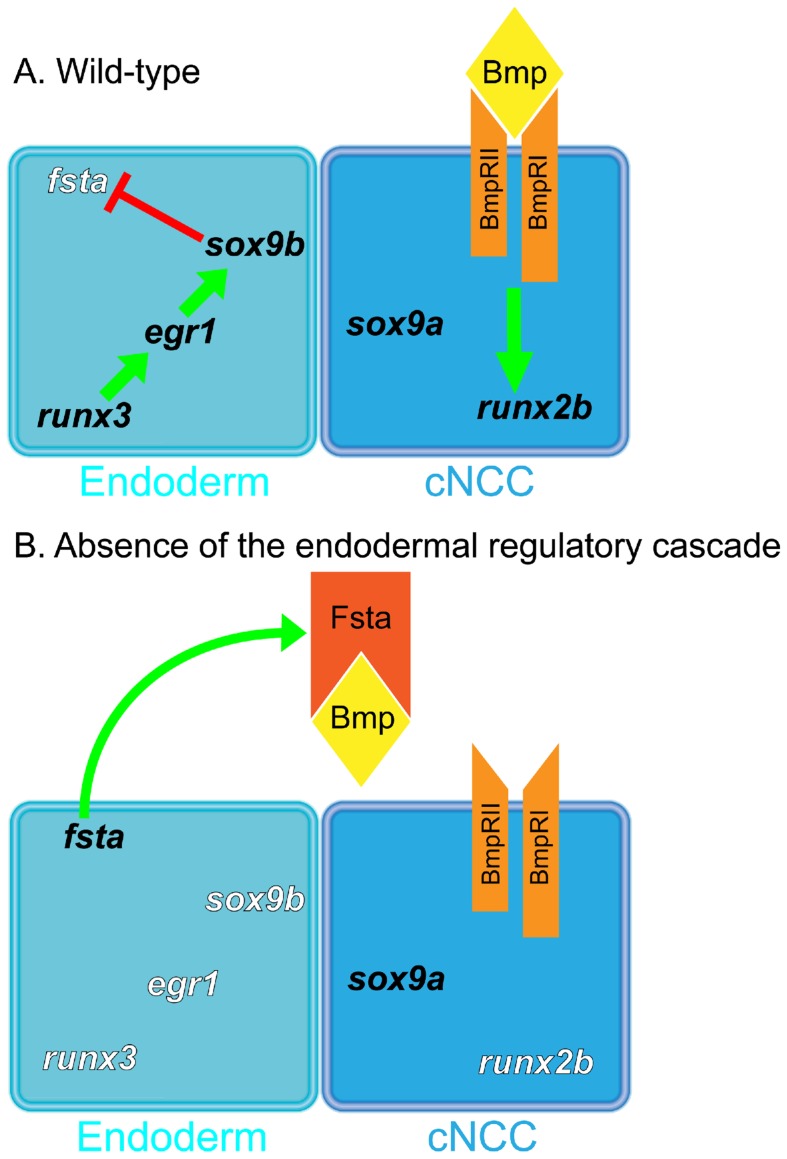
Runx3, Egr1 and Sox9b form a regulatory cascade required to modulate Bmp-signaling during cranial cartilage development in zebrafish. Signaling model in wild-type embryos (A) and in embryos lacking of endodermal regulatory cascade (B). (A) In wild-type embryos, pharyngeal endoderm expresses a regulatory cascade composed of three transcription factors, Runx3, Egr1 and Sox9b, which down-regulates *fsta* expression that codes for a Bmp antagonist. This down-regulation of *fsta* enables Bmp ligands to bind to their heterodimeric receptor (BmpRI and BmpRII) and induce *runx2b* expression in cranial neural crest cells (cNCC). (B) Embryos lacking of any member of Runx3-Egr1-Sox9b cascade have an over-expression of *fsta*, which its coding protein is secreted from the endoderm. Antagonist Fsta binds to Bmp ligands and inhibit them to bind to their receptor, having for consequence no Bmp-signaling towards the cNCC and no *runx2b* expression.

Taken together, our results indicate that the Runx3/Egr1/Sox9b regulatory cascade is active in pharyngeal endoderm after 24 hpf to inhibit expression of *fsta*, which needs to be tightly controlled so that the Bmp ligands can bind to their respective receptors located on cartilage precursors cells (Fig.11A,B). In wild type embryos, BMP signaling also appears to be controlled by the much weaker expression of follistatin A, as illustrated by the presence of increased cartilage elements in *fsta* morphants. BMP action has to be tightly counterbalanced by inhibitory proteins for correct morphogenesis of the head skeleton. Although other BMP antagonists could also contribute to this control, the increased cartilage observed in *fsta* morphants attributes an outstanding role to Follistatin A in this function.

### Multiple Roles of the Pharyngeal Endoderm in Cranial Cartilage Formation

We show that *cas* mutant embryos lack *fsta* expression in the pharyngeal region, as would probably be the case for other mutants devoid of endoderm. However, instead of presenting an increase of cartilage as could be expected, *cas* mutants completely lack a head skeleton and *runx2b* expression at 48 hpf ([46], our own data not shown). This observation obviously reflects the fact that chondrogenic and osteogenic signals (Bmp, Hh, Fgf) originate from the endoderm and thus are absent in the mutants, but it also highlights the importance of maintaining a precise balance of agonistic and antagonistic activities. It further suggests that pharyngeal endoderm may be a major source of BMP signaling in the ventral head region, relative to other surrounding tissues that are still present in the *cas* mutants.

Recently, BMP signaling was shown to be involved at earlier stages (between 17 and 24 hpf) in dorso-ventral patterning by specifying expression of dorso-ventral markers [14]. Here we show that inhibition of BMP signaling by dorsomorphin, expression of a dnBMP receptor or by over-expression of Follistatin A leads to a complete absence of *runx2b* expression, similar to complete absence of endoderm in *cas* mutants [46]. Thus, in addition to early dorso-ventral patterning, BMP signaling is required at later stages for differentiation of all the pharyngeal chondrocytes. Similarly, Shh signaling from pharyngeal endoderm was recently shown to be required before 24 hpf for the selective growth and/or differentiation of anterior cranial cartilage precursor cells, without affecting dorso-ventral patterning, by inducing *fgf8a* expression in oral ectoderm [76]. Ectopic Shha expression by transgenesis in *cas* mutant embryos restored *fgf8a* expression and formation of the mandibular and hyoid arches. Later pharyngeal jaw skeleton was only weakly restored, suggesting the requirement of additional endodermal signals at later stages. According to our results, members of the BMP family coupled to a finely tuned control of Follistatin A levels appear as a good candidate for these signals. The Runx3/Egr1/Sox9b cascade is activated after 24 hpf to reduce *fsta* expression and allow correct BMP signaling that is required at stages beyond 27 hpf for cartilage formation.

Interestingly, manipulation of the endodermal regulatory cascade leaves intact the early function of BMP signaling in dorso-ventral patterning, as indicated by the maintained expression of the ventral markers *hand2* and *edn1* at 24 hpf. This is consistent with the onset of expression after 24 hpf of the regulators Runx3, Egr1 and Sox9b in pharyngeal endoderm and with the observed lack of *fsta* overexpression in *egr1* morphants at 24 hpf. An earlier function for control of BMP signaling by Follistatin A was previously shown in dorso-ventral patterning of the retina [57]. In this case, *fsta* expression was shown to be increased at 13 hpf upon knock-down of the TALE-class homeodomain transcription factor Meis1, leading to a decrease of BMP signaling in the optic vesicle. At 19 hpf, *fsta* expression was highly increased in *meis1* morphants in all its expression domains [57], suggesting a wider role for Meis1 in enabling BMP signaling at this stage. Control of *fsta* expression specifically in the pharyngeal region at later stages is taken over by the endodermal regulatory cascade described here.

### Multiple Signaling Pathways Converge on Developing Chondrocytes

Our results indicate that BMP signaling is required in cNCC cells after their migration to their ventral position in the pharyngeal arches to induce their differentiation into hypertrophic chondrocytes, but also to allow their subsequent migration leading to morphogenesis of the different head cartilages. Additional extracellular signals will also play a role, such as Fgfs or Hhs. At 24 hpf, cNCC cells express the early differentiation marker *sox9a*, whose expression is maintained in pharyngeal cartilage precursor cells and required for their differentiation [6]. Depletion of the endodermal regulatory cascade does not affect *sox9a* expression. In contrast, the key regulatory gene for chondrogenesis and osteogenesis, *runx2b* is dramatically down-regulated in *egr1* morphants. Its expression in pharyngeal cartilage precursor cells normally starts at 34 hpf [46] and was shown to be essential for chondrocyte and osteoblast differentiation [44], [75]. This timing is consistent with the requirement for *fsta* down-regulation for efficient activation by BMP signaling as described here. *runx2b* expression is down-regulated despite the normal expression of *sox9a*, conversely it is not affected in *sox9a* mutants [5], indicating that at least two different pathways are required in chondrocyte precursor cells for activation of these two genes and, thus for chondrogenesis and that neither of them can compensate the function of the other.

Interestingly, inhibition of BMP signaling by increased Follistatin A expression in our experiments led to a decreased expression of *runx2b* in presumptive head cartilage, but left nearly intact its expression in the developing cleithrum, a dermal bone. Similar observations were made in the *sox9a* mutants [5], [6]. These observations indicate that both *sox9a* expression and *fsta* inhibition are mainly required for endochondral (replacement) bone formation.

In conclusion, we show here that a regulatory cascade is active in pharyngeal endoderm that represses expression of the *fsta* gene, thereby allowing the correct activation of BMP signaling in cNCC cells required for their differentiation and morphogenesis of pharyngeal and basicranial cartilage. This cascade starts by increased expression of *runx3*, followed by activation of *egr1* expression and finally *sox9b* expression in the pharyngeal endoderm.

## Supporting Information

Figure S1 ***casanova*** **mutants, lacking endoderm, do not express e** ***gr1*** **at 48 hpf.** Single *in situ* hybridization for *egr1* in *casanova* mutants. Lateral (A,B) and dorsal (C,D) views, anterior to the left. Scale bars 100 µm. (A,C) Wild-type or heterozygous *cas^+/−^* express e*gr1* in the pharyngeal endoderm (pe). (B,D) Homozygous *cas^−/−^* do not express e*gr1* in the pharyngeal region, but in the pectoral fins and in the two hearts of *cas^−/−^* embryos. Pectoral fin (pf), heart (h).
(TIF)

Figure S2 ***casanova*** **mutants, lacking endoderm, do not express** ***sox9b*** **or** ***fsta*** **at 48 hpf.** Lateral views of *in situ* hybridizations (A–D) with indicated markers, anterior to the left. Scale bars 100 µm. Homozygous *cas^−/−^* do not express *fsta* (A,B) nor *sox9b* (C,D) compared to wild-type or heterozygous *cas^+/−^*. Pharyngeal endoderm (pe), otic vesicle (ov).
(TIF)

Movie S1 **Double fluorescent** ***in situ*** **hybridization for** ***fli1*** **(Cy3, red) and e** ***gr1*** **(FITC, green) in 47 hpf zebrafish embryos.** Lateral view, anterior to the left, the movie proceeds from the left side going to the center of the embryo. *fli1* is expressed in cranial neural crest cells (pharyngeal cartilage precursor cells) and in endothelium. *fli1* displays the characteristic expression pattern in “stripes” of the pharygeal cartilage precursor cells. *egr1* is expressed more inside of the embryo in the pharyngeal endoderm. *fli1* and *egr1* mRNAs never colocalize.
(MP4)

Movie S2 **Double fluorescent** ***in situ*** **hybridization for** ***sox9a*** **(Cy3, red) and e** ***gr1*** **(FITC, green) in 72 hpf larvae.** Lateral view, anterior to the right, the movie proceeds from the right side going to the center of the embryo. *sox9a* is expressed in pharyngeal cartilage cells and *egr1* is expressed in pharyngeal endoderm. *sox9a* and *egr1* mRNAs never colocalize and the e*gr1* expression domains surrounding the s*ox9a* expression domains can be clearly observed.
(MP4)

Movie S3 **Double fluorescent** ***in situ*** **hybridization for** ***sox9b*** **(Cy3, red) and e** ***gr1*** **(FITC, green) at 72 hpf.** Lateral view, anterior to the right, the movie proceeds from the right side going to the center of the embryo. *sox9b* and *egr1* are expressed in pharyngeal endoderm cells and colocalize in the same expression domains.
(MP4)

Movie S4 **Double fluorescent** ***in situ*** **hybridization for** ***fli1*** **(Cy3, red) and e** ***gr1*** **(FITC, green) at 4 dpf.** Lateral view, anterior to the right, the movie proceeds from the right side going to the center of the embryo. *fli1* is expressed in pharyngeal cartilages and in endothelium. *Egr1* is expressed in the phrayngeal endoderm and oral epithelium. *fli1* and *egr1* mRNAs never colocalize.
(MP4)
