# Supplementary figures and images for: RUNX3, EGR1 and SOX9B Form a Regulatory Cascade Required to Modulate BMP-Signaling during Cranial Cartilage Development in Zebrafish
Source: PLoS One. 2012 Nov 27;7(11):e50140. doi: 10.1371/journal.pone.0050140 (PMC3507947; doi:10.1371/journal.pone.0050140)

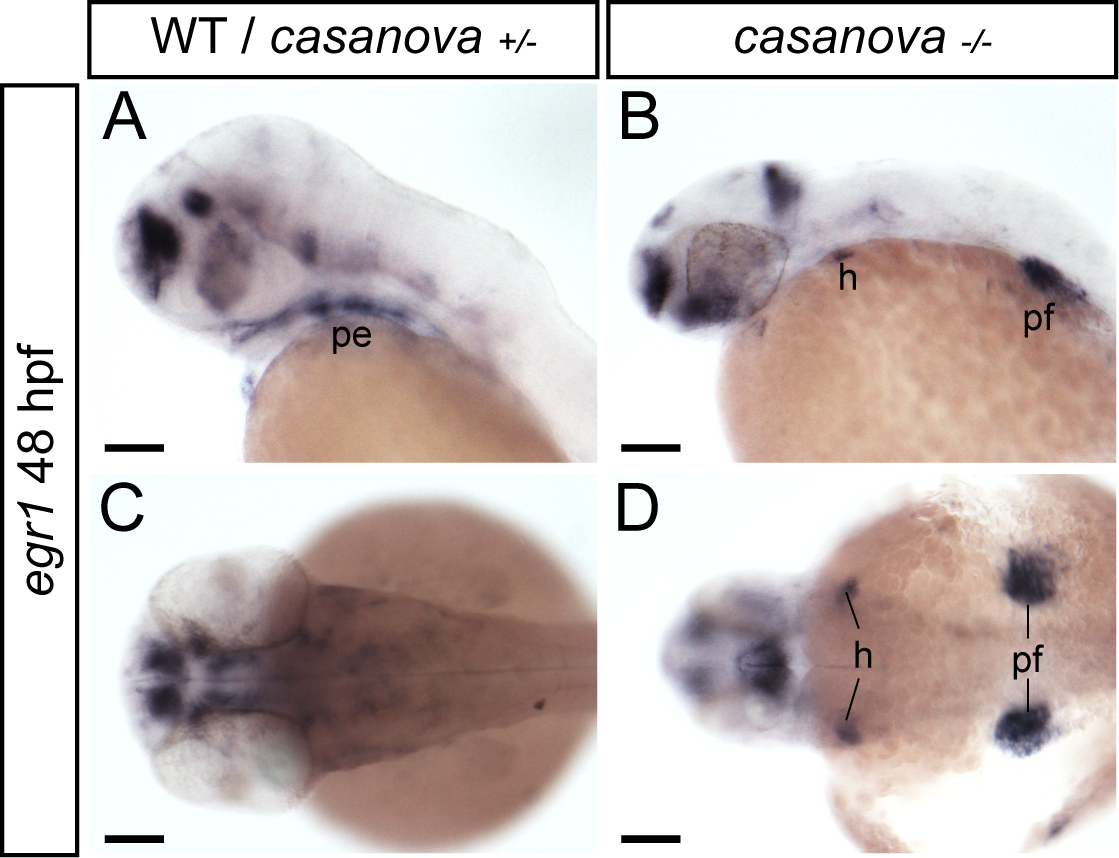

Supplement: Figure S1 — casanova mutants, lacking endoderm, do not express e gr1 at 48 hpf. Single in situ hybridization for egr1 in casanova mutants. Lateral (A,B) and dorsal (C,D) views, anterior to the left. Scale bars 100 µm. (A,C) Wild-type or heterozygous cas+/− express egr1 in the pharyngeal endoderm (pe). (B,D) Homozygous cas−/− do not express egr1 in the pharyngeal region, but in the pectoral fins and in the two hearts of cas−/− embryos. Pectoral fin (pf), heart (h). (TIF) [file pone.0050140.s001.tif]

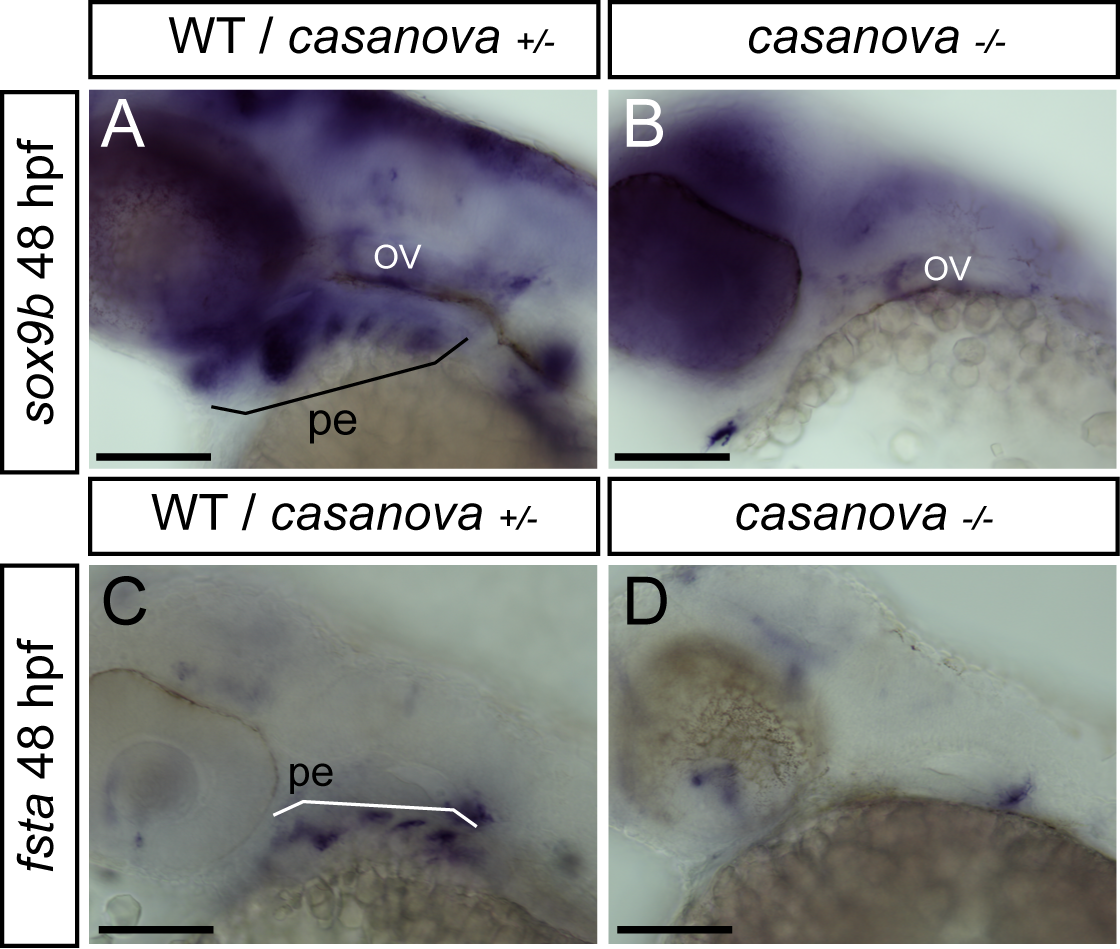

Supplement: Figure S2 — casanova mutants, lacking endoderm, do not express sox9b or fsta at 48 hpf. Lateral views of in situ hybridizations (A–D) with indicated markers, anterior to the left. Scale bars 100 µm. Homozygous cas−/− do not express fsta (A,B) nor sox9b (C,D) compared to wild-type or heterozygous cas+/−. Pharyngeal endoderm (pe), otic vesicle (ov). (TIF) [file pone.0050140.s002.tif]
